# Brain Formaldehyde is Related to Water Intake behavior

**DOI:** 10.14336/AD.2016.0323

**Published:** 2016-10-01

**Authors:** Ting Li, Tao Su, Yingge He, Jihui Lu, Weichuan Mo, Yan Wei, Rongqiao He

**Affiliations:** ^1^State Key Laboratory of Brain and Cognitive Science, Institute of Biophysics, Chinese Academy of Sciences, Beijing 100101, China; ^2^Alzheimer’s Disease Center, Beijing Institute for Brain Disorders, Capital Medical University, Beijing 100069, China; ^3^Key Laboratory of Mental Health, Institute of Psychology, Chinese Academy of Sciences, Beijing 100101, China; ^4^Southwest Medical University, Sichuan 646000, China; ^5^Beijing Geriatric Hospital, Beijing 100095, China; ^6^University of Chinese Academy of Sciences, Beijing 100049, China

**Keywords:** formaldehyde, water intake, semicarbazide-sensitive amine oxidase, arginine vasopressin, behavior

## Abstract

A promising strategy for the prevention of Alzheimer’s disease (AD) is the identification of age-related changes that place the brain at risk for the disease. Additionally, AD is associated with chronic dehydration, and one of the significant changes that are known to result in metabolic dysfunction is an increase in the endogenous formaldehyde (FA) level. Here, we demonstrate that the levels of uric formaldehyde in AD patients were markedly increased compared with normal controls. The brain formaldehyde levels of wild-type C57 BL/6 mice increased with age, and these increases were followed by decreases in their drinking frequency and water intake. The serum arginine vasopressin (AVP) concentrations were also maintained at a high level in the 10-month-old mice. An intravenous injection of AVP into the tail induced decreases in the drinking frequency and water intake in the mice, and these decreases were associated with increases in brain formaldehyde levels. An ELISA assay revealed that the AVP injection increased both the protein level and the enzymatic activity of semicarbazide-sensitive amine oxidase (SSAO), which is an enzyme that produces formaldehyde. In contrast, the intraperitoneal injection of formaldehyde increased the serum AVP level by increasing the angiotensin II (ANG II) level, and this change was associated with a marked decrease in water intake behavior. These data suggest that the interaction between formaldehyde and AVP affects the water intake behaviors of mice. Furthermore, the highest concentration of formaldehyde in vivo was observed in the morning. Regular water intake is conducive to eliminating endogenous formaldehyde from the human body, particularly when water is consumed in the morning. Establishing good water intake habits not only effectively eliminates excess formaldehyde and other metabolic products but is also expected to yield valuable approaches to reducing the risk of AD prior to the onset of the disease.

As described by Luckey and Parsa, many people do not maintain water homeostasis as they age [1]. Indeed, water represents 60% of the body mass of young men, whereas in aging people, this contribution is reduced to 50%. In non-pathological conditions, aging is accompanied by a progressive decrease in the water balance (due to reductions in the feeling of thirst and water intake) that is suspected to be the cause of a reduction in general body hydration [2].

Weight loss is also a major clinical feature of Alzheimer’s disease (AD). According to Purdy [3], acceleration in the rate of weight loss is a harbinger of the change from a non-demented status to Alzheimer’s-type dementia. Weight loss primarily results from chronic dehydration, which may be one of the preventable risk factors for Alzheimer’s disease [4]. Chronic dehydration is regarded as a common symptom of patients with age-related cognitive impairment, particularly those with AD [5, 6]. Alzheimer’s disease is characterized by a tendency to exhibit malnutrition, which is present even in the mild-moderate stages, and a tendency to exhibit dehydration that appears in the severe stage [7]. Hyperosmotic stress induces apoptosis and Tau phosphorylation in human neuroblastoma cells [8, 9, 10]. Greater weight loss is associated with increased disease severity and mortality [11]. However, the inducers that are involved in chronic dehydration require further attention. Recently, formaldehyde (FA), which is present in all human cells including neural cells, was found to affect age-related cognitive impairment [12, 13, 14]. The levels of uric FA are positively related to the severity of cognitive impairment in clinical AD patients [15]. Intravenous injections of FA into the tail lead to Tau hyperphosphorylation in wild-type mouse brains through the activation of glycogen synthase kinase-3β (GSK-3β) [16]. The oral administration of methanol (the metabolic precursor of FA) triggers the formation of senile plaques (SPs) and Tau hyperphosphorylation in the brains of monkeys and is accompanied by a decrease in working memory [17, 18]. Formaldehyde-induced Tau aggregation has been implicated in neuronal cytotoxicity and neural cell apoptosis [19, 20]. Furthermore, FA also affects the emotional behaviors of animals, including depression and anxiety [21, 22]. Although the relationship between FA and brain dysfunction has been intensively studied [23, 24], the effects of formaldehyde on water intake behavior and dehydration have not been investigated to date.

Arginine vasopressin (AVP) is known to be involved in the water intake behaviors of animals [25, 26, 27]. AVP and aldosterone (ALD) are secreted by the neurohypophysis in response to increased angiotensin II (ANG II) levels [28] and blood osmolality [29, 30, 31]. AVP increases peripheral vascular resistance and exerts antidiuretic effects that promote water reabsorption in the collecting ducts of the kidney [32, 33, 34]. Due to their low serum AVP levels, mice with a familial ALS-associated SOD1 mutation exhibit significantly increased water intake compared with wild-type mice [35]. The endogenous formaldehyde concentration is known to increase with aging [15]. However, whether formaldehyde’s effects on serum AVP levels are associated with water intake behavior remains unknown.

This paper is concerned with the relationship between increased endogenous formaldehyde levels and water intake behaviors with aging. We show that both the endogenous formaldehyde and serum AVP levels increase with aging. The increased formaldehyde levels promote AVP expression by activating ANG II. AVP administration increases the endogenous formaldehyde levels by activating semicarbazide-sensitive amine oxidase (SSAO). The pathways create a vicious cycle that affects water intake behavior and results in decreased drinking frequency and water intake.

## MATERIALS AND METHODS

### Animals and rearing environment

C57 BL/6 mice (male, 3-, 6- and 10-months old) were provided and maintained by the Animal Experiment Center of the Institute of Biophysics (IBP) of the Chinese Academy of Sciences (CAS). Three mice were reared in each “shoebox” cage. The temperature and humidity in the experimental room were maintained at 22±2 °C and 40%-60%, respectively. All of the experiments were performed after the animals had been acclimated to the room for 7 days. The animals’ body weights were measured before the experiments. All animal experiments were approved by the Animal Care and Use Committee at the IBP of the CAS (Authorization No.: SYXK2013-77).

### Animal water intake tests

The drinking behaviors of the mice were recorded with infrared CCD cameras affixed to the ceiling as previously described [36]. Before the test, the animals were allowed to acclimate to the test environment for 2 h. Drinking attempts were recorded as each occasion that the animal licked the bottle tip for more than two seconds. The drinking frequencies and water intake attempts were determined in counts per hour over three continuous days. In the AVP-injected group, the drinking frequencies and water intake attempts were determined in counts per hour from 8:00 p.m. (the first day) to 8:00 p.m. (the second day). No animals were injured or died during the experiment. Additionally, the volumes of water consumed were determined by measuring the volumes in the cylinders over three continuous days.

### Assays of the AVP and ANG II levels in the serum and SSAO levels in the mouse brain

The mice were sacrificed, and blood samples were collected by eyeball enucleation as described by Wu and colleagues [37]. The serum samples were collected by centrifugation (4,000 r/min, 25 °C, 15 min, TDZ5-WS, Xianyi, Hunan, China). The serum osmotic pressure was determined using an osmometer (OM806, LOSER, Germany). Next, the samples were stored at -80°C until they were assayed. The serum ANG II, AVP, and SSAO levels and activities were detected with ELISA kits (TSZ, Shanghai, China) according to the manufacturer’s instructions. Note that the brain tissues were freshly and quickly dissected to measure the SSAO concentrations and activities.

### Assay of the mouse brain formaldehyde levels by UV-HPLC

The brain (0.1 g) was homogenized in 0.5 ml of SDN lysis buffer immediately after the mouse was sacrificed. Next, 0.5 ml of 10% trichloroacetic acid (analytical purity, Xilong Chemical Co., Ltd., USA) was added prior to centrifugation (12,000 rpm, 4°C, 30 min). The supernatants (0.4 ml) were pipetted into 1.5-ml Eppendorf tubes and mixed with 0.5 ml acetonitrile (HPLC-grade purity, Fisher Scientific, USA) and 0.1 ml 2,4-dinitrophenylhydrazine (DNPH, analytic purity, Beijing Chemical Reagent Research Institute, China). Next, the samples were centrifuged (12,000 rpm, 4°C, 10 min), incubated in a 60°C water bath for 30 min, and centrifuged again (12,000 rpm, 4°C, 10 min). The formaldehyde levels were determined with HPLC as described by Su and coworkers [38].

### Intraperitoneal injection of formaldehyde and water intake recordings in the mice

As previously described [39], 3-month-old mice were intraperitoneally injected with formaldehyde (0.5 mg/kg, once daily) for 7 days. Then, their drinking frequencies (counts/hour) and water intake quantities (ml/hour) were continuously recorded with an infrared CCD camera for 3 days. Subsequently, their brain formaldehyde and serum ANG II and AVP levels were determined as described above. Mice that were injected with saline placebo were used as controls.

### Open field test following the intraperitoneal injection of formaldehyde

After treatment with FA as described above, the mice were subjected to an open field test in an apparatus that consisted of a 40×40-cm open arena with 30-cm-high walls (Huaibeizhenghua Biological Instrument Equipment Co., Ltd., China) [37]. The entire test arena was adjusted to ensure even illumination. The mice were placed in the center of the arena, and their activities were recorded for 5 min. For the video analysis, the open field arena was divided into 16 equal squares, and the 4 center squares were defined as the central zone. The numbers of square crossings (times), grooming (times), vertical frequency (times) and the time spent in the central square (seconds) were recorded.

### Intravenous injection of AVP in the tail and water intake behavior recordings in the mice

Three-month-old mice were subjected to intravenous injections of AVP (0.2 ng/kg) through the tail as previously described [40]. After the injection, the drinking frequencies and quantities of water consumed by the mice in each group were recorded at different times (0 h, 0.5 h, 1 h, 2 h, 4 h, 12 h, and 24 h), and the serum AVP and brain formaldehyde concentrations and enzymatic activity of brain SSAO were subsequently measured as described above. Mice injected with the saline placebo were employed as controls.

### Participants recruited for the water intake trial with normal young people

This human trial was approved by the Ethics Committee at IBP, CAS (2015-HRQ-1). The study has been registered at the Chinese Clinical Trial Registry (ChiCTR) with the unique identifier ChiCTR-IPC-15005812 (www.chictr.org.cn). Twenty volunteers (25-35 years old, 9 males and 11 females) were recruited to participate in this trial. Each volunteer provided informed consent before participating in the trial. To guarantee the quality of this trial, the candidates participated in physical examinations. Any candidate who was unhealthy was excluded before the trial began. The examinations revealed that there were no abnormal results in the volunteers’ alanine transaminase (ALT), blood glucose (GLU), urea, urinary creatinine, uric acid (UA), triglyceride (TG), total cholesterol (TC), high density lipoprotein (HDL) or low density lipoprotein (LDL) levels (personal data not shown; Supplementary Table 1). These findings demonstrated that all of the enrolled participants were in normal health and without any hepatic or renal disorders. Their body weights did not significantly change during the trial (Supplementary Table 2).

Food was provided by the authors. All participants ate uniform meals during each day of the trial (Supplementary Table 3) [41]. The participants were prohibited from eating food other than the meals provided and drinking any beverage (e.g., coffee, milk, honey, and tea) other than the water provided. The participants were instructed to go to sleep at 11:00 in the evening and arise at 7:00 in the morning. All participants performed indoor work in the laboratory during the day. When they were not working, they were not allowed to attend other activities, such as physical exercises, shopping or dating, during the trial. Anyone who violated these instructions, including staying up all night, was excluded from the trial.

### Recording water intake and the uric formaldehyde levels in the normal young people

The trial was divided into the following three steps: 1) the participants drank water according to their usual water intake habits regarding both drinking frequency and water quantity, 2) water deprivation (from 8:30 in the morning after breakfast until 12:30 in the afternoon before lunch), and 3) water intake prescribed according to the Chinese DRIs Handbook provided by the China Nutrition Association [41]. For the water intake control group, averages of 500 ml of water for males and 400 ml of water for females were provided from 8:30 in the morning after breakfast until 12:30 in the afternoon before lunch. Commercial mineral water from the supermarket was provided for the participants. Each participant was required to consume all of the water, but additional water was not administered when a participant wanted to drink more.

During the trial, each participant provided urine samples according to the authors’ instructions. Mid-stream urine (5 ml) was first sampled in the morning (7:00) before breakfast, a second urine sample was provided before lunch (12:30), and the last sample was sampled before going to sleep at approximately 11:00 p.m. The samples were placed in a refrigerator (4°C) before use. A urine sample (1 ml) was placed into a centrifuge tube and centrifuged (12,000 rpm, 4°C, 30 min), and the supernatant was used to measure the formaldehyde levels with HPLC as described above. According to the methods of Wang and colleagues [42], the urine creatinine (Urc) level was measured using a kit (Bi Yuntian, Beijing) to normalize the formaldehyde measurements. The concentrations of uric formaldehyde are presented as the [FA]/[Urc] ratios unless otherwise stated.

### Participants with cognitive impairments were recruited from patient clinics

This trial was approved by the Ethics Committee at IBP, CAS (2015-HRQ-1). The study is registered at ChiCTR with the unique identifier ChiCTR-IPC-15005812 (www.chictr.org.cn). Patients with Alzheimer’s disease (n = 62, 81.05 ± 6.36 years old) and normal elderly (n = 69, 67.33 ± 6.19 years old) participants were randomly recruited from the Memory Clinic of Beijing Geriatric Hospital and a nearby community, respectively, from March 2015 to April 2015. Alzheimer’s disease was diagnosed according to the criteria for probable AD defined by the National Institute of Neurological Disorders and Stroke-Alzheimer’s Disease and Related Disorders Association (NINCDS-ADRDA). The participants were considered to be cognitively normal if they scored 0 on the Clinical Dementia Rating (CDR) scale and did not complain of cognitive dysfunction. All diagnoses were determined by a team of at least 3 experienced dementia specialists from the Beijing Geriatric Hospital [43]. Participants with abnormal liver function, abnormal renal function, abnormal blood sugar levels, or other comorbid conditions were excluded from the study. The cognitive functions of all participants in both the AD and normal control groups were examined with the MMSE, and the results are presented in Supplementary Table 4A. To guarantee the reproducibility of the analytical results, the morning urine samples were collected before the participants had breakfast. The participants were asked to eat their normal diet and avoid consuming fatty and spicy foods for one week prior to sampling. The samples were stored in sealed sterile containers at -80°C at the Beijing Geriatric Hospital until they were sent to the IBP for double-blind analyses. The urine creatinine (Urc) was measured using a kit (Bi Yuntian, Beijing) to normalize the formaldehyde measurements.

### Data analysis

The quantitative and categorical data were compared using analyses of variance (ANOVAs), Fisher’s exact tests, and nonparametric tests. The statistical analyses were conducted with SPSS 17.0 (International Business Machines Corporation, USA).

## RESULTS

### Increased brain formaldehyde levels and decreased water intake with aging

**Figure 1.**
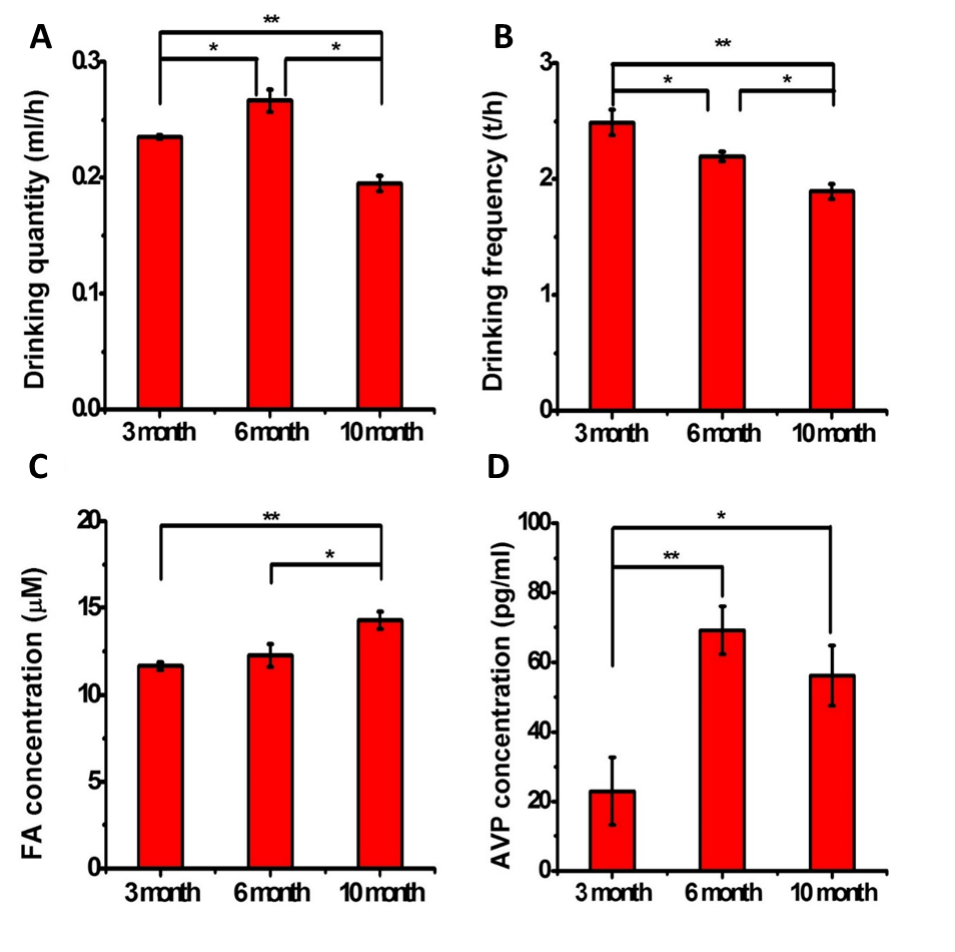
**Changes in quantity and frequency of water intake as well as the brain formaldehyde and AVP concentrations in mice at different ages**. C57 BL/6 mice *were* maintained *under* pathogen-free *conditions* (22 ± 2°C, humidity 50%) and provided a regular diet and sterile water. Their drinking behaviors were *recorded* with an infrared camera, followed by counting the volume (ml/h) (**A**) and frequency (times/h) of water intake (**B**) at different ages (3, 6 and 10 months, n = 6). Their brain formaldehyde (μM) (**C**) and blood AVP (pg/ml) (**D**) levels were also measured at these ages (n = 8). The data are shown as the means ± SE; *, *P* < 0.05; **, *P* < 0.01

To investigate the relationship between the formaldehyde levels and water intake behavior, wild-type C57 BL/6 mice were fed a regular diet and housed under pathogen-free conditions, and the brain formaldehyde concentrations were determined at different ages (3, 6 and 10 months). Simultaneously, the animals’ water intake behaviors were recorded with an infrared CCD camera. The water intake quantities (ml/hour) significantly increased from 3 to 6 months (n = 6, *P*<0.05) and decreased at 10 months (n = 6, *P*<0.01). The water intake quantity of the 10-month-old mice was less than those of both the 3- and 6-month-old mice (Fig. 1a). The drinking frequency (counts/hour) of the 10-month-old mice was significantly decreased compared with those of both the 3-month-old (n = 6, *P* < 0.01) and 6-month-old mice (n = 6, *P* < 0.05; Fig. 1B). These data demonstrated that the water intakes of the mice decreased with age.

Moreover, we observed changes in the brain formaldehyde concentrations in the mice at different ages as illustrated in Fig. 1C. The brain formaldehyde levels of the 10-month-old mice were significantly increased compared with those of the 3-month-old (n = 8, *P* < 0.01) and 6-month-old mice (n = 8, *P* < 0.05). Therefore, the endogenous formaldehyde concentrations increased with age. To investigate whether the decreased water intake was related to the endogenous formaldehyde levels, the mice were deprived of water for 3 days, and their brain formaldehyde levels were then measured. The results revealed that water deprivation caused a significant increase in the brain formaldehyde levels compared with the control group (Supplementary Fig. 1).

**Figure 2.**
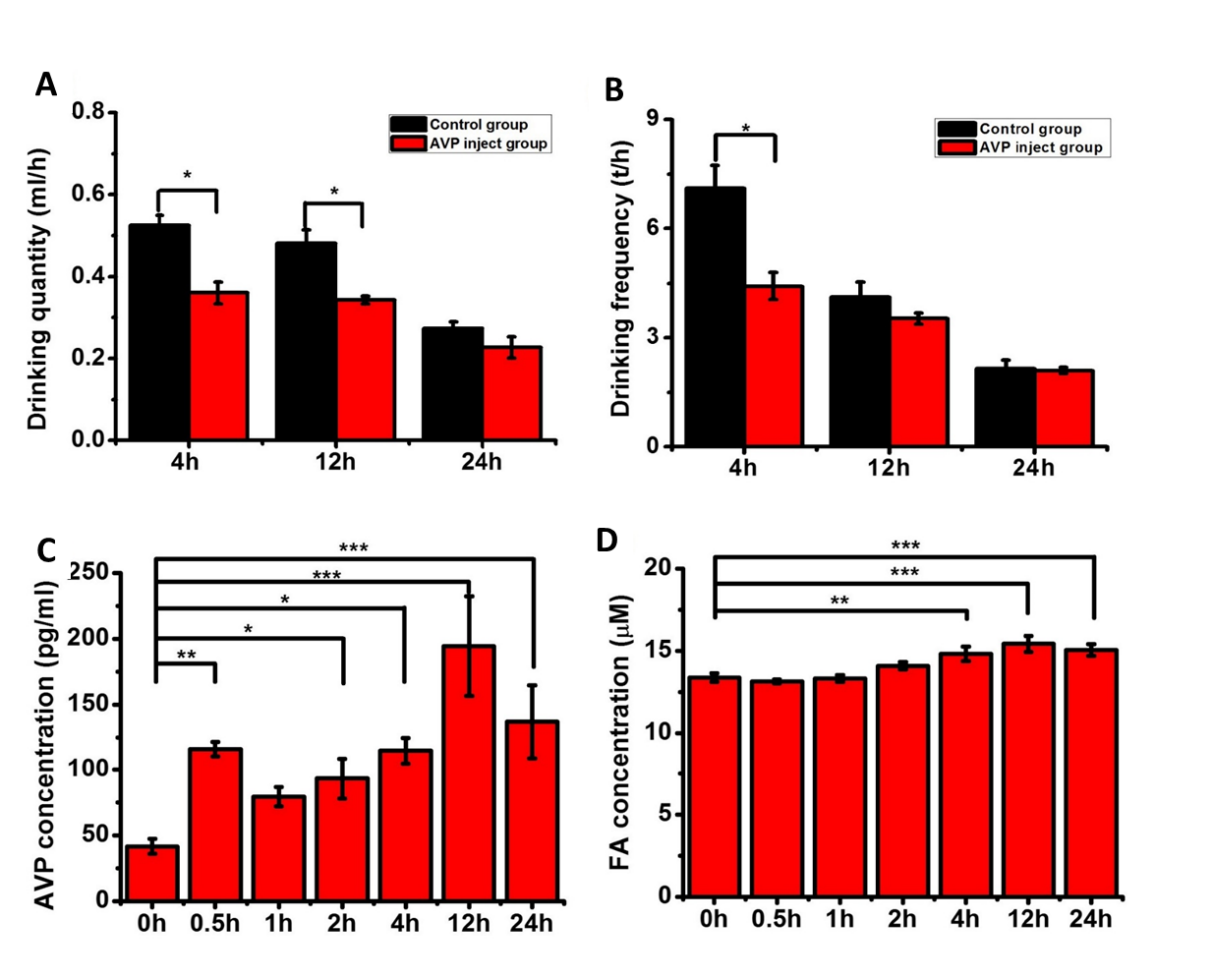
**Changes in the water quantities, drinking frequencies, and brain formaldehyde and serum AVP concentrations in mice after the AVP injection**. The conditions were the same as Figure 1, except that the 3-month-old C57 mice (n = 8) were administered 100 μl of AVP (2 ng/kg, once at the beginning) through an intravenous injection in the tail, followed by measurements of their drinking quantities (**A**), frequencies (**B**), and brain formaldehyde (**C**) and AVP levels (**D**) for 24 hours. The data are shown as the means ± SE; *, *P* < 0.05; **, *P* <0.01; ***, *P* < 0.001.

According to Takeuchi and colleagues [44], AVP is involved in water intake behavior. To investigate why the mice naturally decreased their water intakes with age, we measured the serum AVP concentrations in the mice at different ages. As illustrated in Fig. 1D, the 6-month-old and 10-month-old mice exhibited higher serum AVP concentrations compared with the 3-month-old mice, but there was no significant difference (P = 0.296) between the 6-month-old and 10-month-old mice. During the experiments, the body weights of the 10-month-old mice markedly increased (*P* < 0.05; Supplementary Fig. 2A), but there was no significant difference in their serum osmolalities (*P* > 0.05; Supplementary Fig. 2B). The high levels of AVP appeared to play a role in the decreased water intake of the elderly mice.

### AVP injection induces changes in water intake behavior

To demonstrate that the changes in the AVP levels were associated with water intake behavior, we intravenously injected 3-month old mice with AVP through the tail to observe whether increased AVP levels altered their water intake behavior. As illustrated in Fig. 2A, the water intake quantity within 4 hours was significantly (*P*<0.05) decreased for the mice who had been injected with AVP, and this decrease was associated with a decrease in drinking frequency (*P* < 0.05), compared with the values of the control group (Fig. 2B). Note that the AVP injection increased the mouse serum AVP concentration (Fig. 2C). Therefore, the increased serum AVP level resulted in decreased water intake behavior in the mice in our experimental conditions.

### AVP increases brain formaldehyde levels by activating SSAO

**Figure 3.**
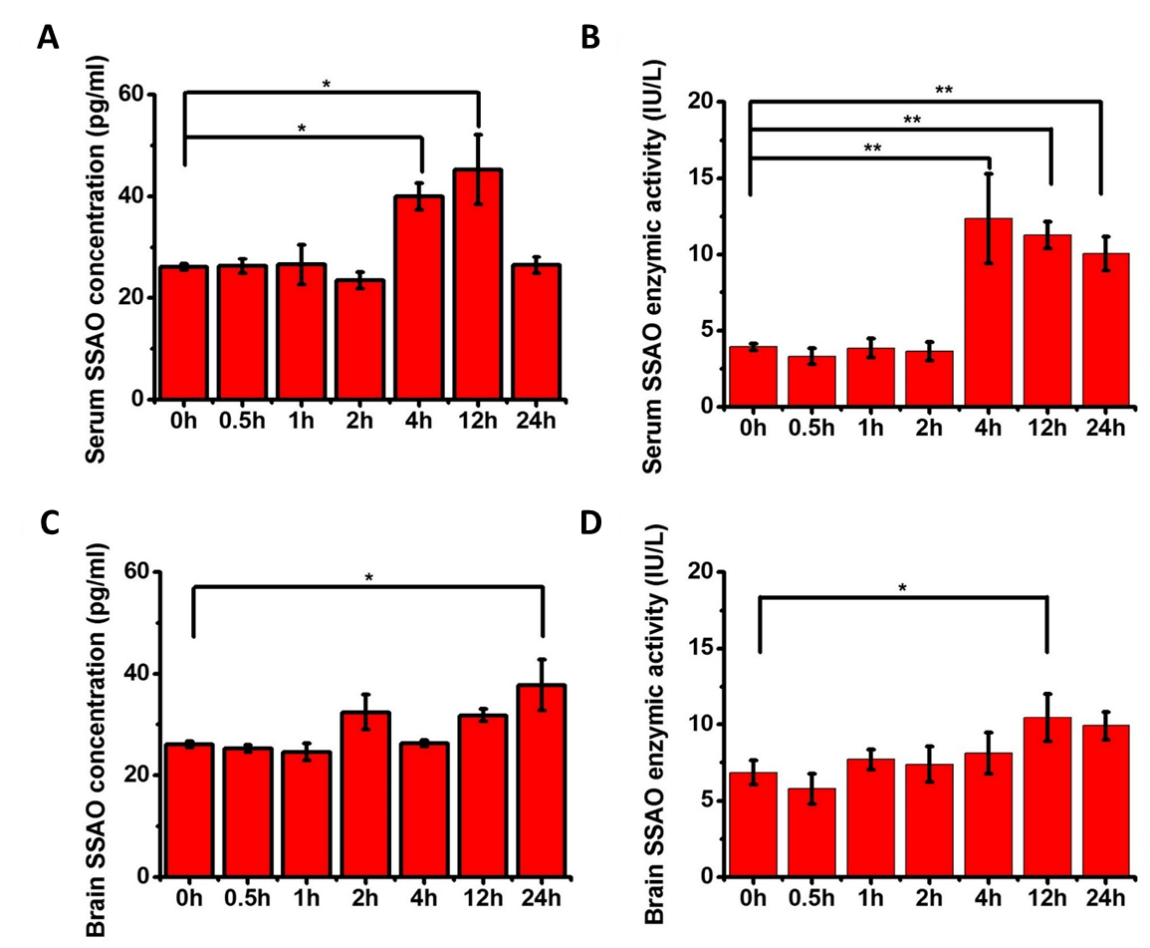
**Concentrations and enzymatic activities of SSAO in the brain and serum of mice injected with AVP**. The conditions were the same as for Figure 2. After an intravenous injection of AVP in the tail, we detected the concentrations and enzymatic activities of SSAO in the serum (**A, B**) and brain (**C, D**) by ELISA. The data are shown as the means ± SE; *, *P* < 0.05; **, *P* < 0.01; ***, *P* < 0.001.

As illustrated in Fig. 2D, the AVP injection markedly elevated the brain formaldehyde levels from 4 h to 24 h (n = 8, *P* < 0.001). To clarify the mechanism responsible for the elevated formaldehyde levels, we detected the changes in both the level and activity of the SSAO protein. An ELISA with an anti-SSAO antibody revealed that the levels of the SSAO protein were increased in the sera (n = 8, *P* < 0.05) 4 hours after the administration of AVP (Fig. 3A), and this increase was followed by an increase in the SSAO activity (n = 8, *P*<0.01) as measured with the ELISA kit (Fig. 3B). Furthermore, we also detected the level (Fig. 3C) and activity (Fig. 3D) of the SSAO protein in the mouse brain and found that both increased following AVP injection (n = 8, *P*<0.05). These data indicated that AVP increased the endogenous formaldehyde level by activating SSAO.

**Figure 4.**
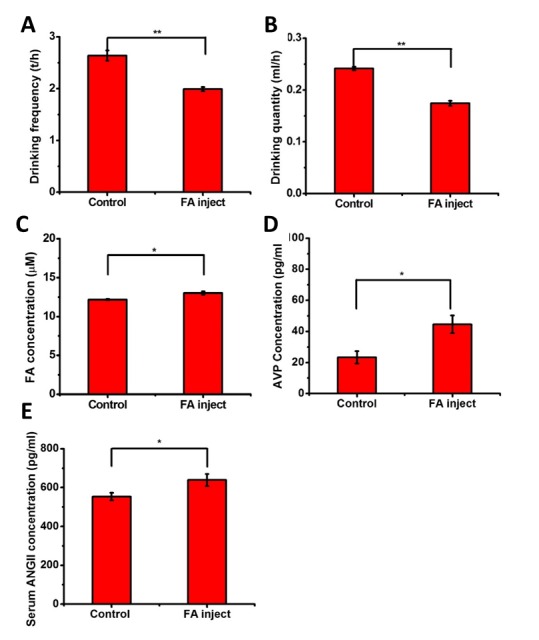
**Changes in the water quantities, drinking frequencies, and brain formaldehyde and serum AVP concentrations in mice injected with formaldehyde**. The conditions were the same as Figure 1, except that the 3-month-old C57 mice (n = 8) were intraperitoneally injected with formaldehyde (0.5 mg/kg, once daily) for 7 days, followed by measurements of their drinking frequencies (**A**), water quantities (**B**), and brain formaldehyde (**C**), serum AVP (**D**) and ANG II levels (**E**). The data are shown as the means ± SE; *, *P* < 0.05; **, *P* < 0.01.

### The animals’ water intakes decreased following injection with formaldehyde

To investigate whether formaldehyde affected water intake, we intraperitoneally injected the mice with formaldehyde (0.5 mg/kg, once daily) for 7 days. Both the quantity and frequency of water intake were significantly (*P* <0.01) decreased after the mice (n = 8) were injected with formaldehyde (Fig. 4A, 4B). A distinct increase in the brain formaldehyde level (*P* < 0.05, Fig. 4C) was be detected and was, which accompanied by an increase in the serum AVP level (*P*<0.05, Fig. 4D). Further determinations revealed observable increases in body weight (*P* = 0.056) and the serum osmolality (*P* = 0.092; Supplementary Fig. 3A and 3B). These data suggest that formaldehyde influences the water intake behaviors of mice by increasing the AVP levels.

ANG II is known to positively regulate both AVP and ALD [25]. Thus, we measured the concentration of ANG II after the mice were injected with formaldehyde. As illustrated in Fig. 4E, the administration of formaldehyde significantly increased the serum ANG II concentration (n=8, P<0.05). As a result of the increased angiotensin levels, a significant increase in the serum ALD (P=0.03) was also detected (Supplementary Fig. 3C). This result suggests that formaldehyde promotes AVP and ALD expression by increasing the serum ANG II level.

**Figure 5.**
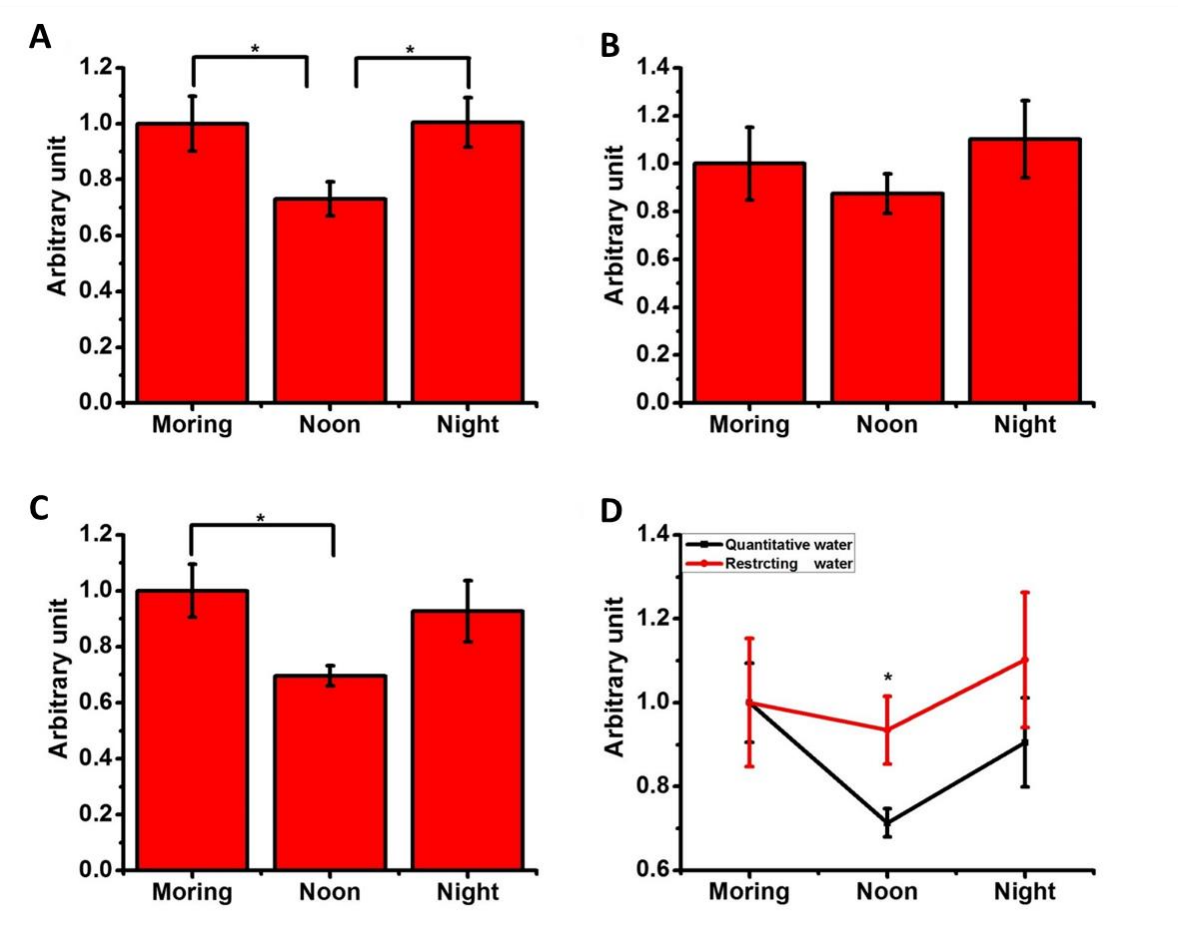
**Changes in the concentrations of formaldehyde in humans subjected to water deprivation**. The participants’ (n = 20) activities were prescribed as indicating in the recruiting requirements. Urine samples were collected in the morning before breakfast (8:00 a.m.), prior to lunch (12:30) and before going to sleep (11:00 p.m.) to measure the formaldehyde levels (FA/Urc ratios). The changes in the uric formaldehyde concentrations were measured for the participants who drank water according to their own daily habits (**A**), those who were forbidden from drinking water (**B**) and those who drank water in prescribed quantities from 8:30 to 12:30 (**C**). Comparison of the formaldehyde concentrations between the water-deprived group and the group with a prescribed water quantity (**D**). The concentration of formaldehyde in the morning was set to 100%. The data are shown as the means ± SE; *, *P* <0.05; **, *P* <0.01.

To exclude the behavior observed after formaldehyde that resulted from more general effects, we performed an open field test. No significant differences were observed in the numbers of square crossings (Supplementary Fig. 4A) or the vertical frequency (Supplementary Fig. 4B); these findings indicate that locomotion was normal. Similar results were obtained for grooming (Supplementary Fig. 4C) and the time spent in the central square (Supplementary Fig. 4D), which indicates that the mice did not exhibit anxiety-like moods.

### Changes in the endogenous formaldehyde levels and water intake

In the humans, we observed the changes in the uric formaldehyde concentrations to understand formaldehyde metabolism. During the trials, the participants’ diets (n=20) were prescribed according to their body weights. The participants’ demographic characteristics and daily actions were also monitored as described in the Materials and Methods.

To investigate the changes in the urine formaldehyde concentrations, an estimation of the daily water intakes of the participants was required. The daily water intake for Beijing residents averages 1,500 ml (1,679 ml for men and 1,370 ml for women) in addition to water intake from vegetables as reported by Zhang and colleagues [45]. As presented in Supplementary Table 5, the average water intakes of the participants in the morning, afternoon and evening were 375 ml, 358 ml and 333 ml, respectively. The average daily water intakes were 1,244 ml for males and 918 ml for the females during the trial.

First, we recorded the changes in the formaldehyde concentrations of the participants who drank water according to their own habits (Supplementary Table 5). Fig. 5A illustrates that the uric formaldehyde concentrations at noon were significantly decreased compared with those in the morning (*P*<0.05) and evening (*P* <0.05). The concentrations observed in the morning were greater than those observed at noon (*P* <0.05) and before sleep (*P* = 0.248). These findings indicate that endogenous formaldehyde accumulates in our bodies as we sleep.

To investigate whether water intake affects the concentrations of formaldehyde, the participants were deprived of water after breakfast (8:30 a.m.) until their urine samples had been collected before lunch (12:30 p.m.) (Fig. 5B). The noon formaldehyde levels were increased to 7.45 ± 0.725 μM compared with the level of 6.59 ± 0.283 μM observed in the group whose water intake was prescribed according to the Chinese DRIs Handbook (*P* < 0.01). The accumulation of formaldehyde at noon was not significantly different from the levels observed in the morning (*P* = 0.337) or evening (*P* = 0.086). Therefore, water deprivation leads to the accumulation of endogenous formaldehyde in our bodies.

To clarify whether water intake aid the elimination of endogenous formaldehyde, we regulated the participants’ water intakes according to the recommendations of the Chinese DRIs Handbook [41] to observe the daily changes in their uric formaldehyde concentrations (Fig. 5C). Similar changes in the formaldehyde concentrations were observed in the participants with regulated water intake and those with free access to water according to their own habits. The concentrations of formaldehyde in the morning (*P* = 0.985), at noon (*P* = 0.703) and in the evening (*P* = 0.993) in the regulated water intake group were not significantly different from those in the group with ad libitum water intake. However, the concentration of formaldehyde in the regulated water intake group was significantly different from that of the group that was subjected to water deprivation at noon (P < 0.05, Fig. 5D). Therefore, the endogenous formaldehyde concentration significantly decreased at approximately noon compared with the concentration in the morning when participants regularly drank water after breakfast. Good water intake habits are thus important and beneficial to the prevention of the accumulation of endogenous formaldehyde during the day.

### Endogenous formaldehyde levels were significantly increased in the Alzheimer’s patients

As previously described [15], endogenous formaldehyde concentrations increase with age (over 75 years old). Here, we recruited 62 AD patients and 69 normal participants (Supplementary Table 6 and 7) for this project. Morning urine samples were collected to measure the endogenous formaldehyde concentrations (Supplementary Table 8). The uric formaldehyde levels of the AD participants were significantly greater (*P* < 0.01) than those of the normal participants (Supplementary Fig. 5A). Similar results were also observed when the uric formaldehyde concentrations of the male (*P* <0.05, Supplementary Fig. 5B) and female AD patients were compared with those of the normal participants (*P*<0.001, Supplementary Fig. 5C). To differentiate the Alzheimer’s patients from the normal aged participants, we selected 70-80 year-old participants and compared the urine formaldehyde concentrations between the patients and normal participants. Consistently, the data indicated the same trend when the age factor was excluded (Supplementary Table 4b). These data demonstrated that endogenous formaldehyde levels not only increased with aging (over 75 years old) but were also markedly increased in the AD patients.

## DISCUSSION

Chronic dehydration is a common symptom observed in AD patients [4]. This study is the first to clarify the link between formaldehyde and animal water intake behavior. The mice exhibited decreased drinking frequencies and water intake quantities as they aged. Based on this evidence, increased formaldehyde levels are thought to be involved in the chronic dehydration of AD patients because endogenous formaldehyde levels are positively correlated with the severity of the cognitive impairments of AD patients in clinics [13, 46]. The endogenous formaldehyde concentrations of AD patients are much greater than those of age-matched elderly people [15]. According to Munoz and colleagues [47], chronic dehydration is related to cognitive impairment; i.e., cognitive impairment causes patients to forget to drink water, which aggravates their dehydration. Thus, regular water intake should be recommended to eliminate the excess formaldehyde in the early stages of age-related cognitive impairment.

The dysmetabolism of formaldehyde and AVP may form a vicious cycle of chronic dehydration that results from decreased water intake (Supplementary Fig. 6). This perspective is based on the following observations: 1) decreased water intake (in terms of both quantity and frequency) occurs as mice age and is followed by marked increases in the brain formaldehyde and serum AVP levels; 2) the endogenous formaldehyde concentrations in elderly people (over 75 years old) are significantly greater than those in younger people [46]; 3) the endogenous formaldehyde concentrations in AD patients were much greater those in the normal controls; 4) the administration of formaldehyde decreased water intake (in terms of both quantity and frequency) by activating ANG II, which increased the serum AVP concentrations; 5) AVP injections suppressed water intake and increased brain formaldehyde levels by activating SSAO; 6) water deprivation for 3 days led to increased brain formaldehyde and AVP levels in the mice; and 7) endogenous formaldehyde concentrations are positively correlated with the severity of the cognitive impairments of AD patients [48, 49], which suggests that high levels of formaldehyde cause these patients to forget to drink water, which in turn, aggravates their dehydration. Note that increased formaldehyde may act as a stressor that stimulates the hypothalamic-pituitary-adrenal (HPA) axis and elevates the levels of ANG II and AVP, and these elevations could affect the quantity and frequency of water intake in mice [50, 51], particularly following the intraperitoneal injection of formaldehyde.

ANG II and AVP are known to be involved in water intake behaviors [52]. The endocrine system secretes ANG II and AVP, which play important roles in the modulation of neurohypophyseal secretions, ANG II production and thirst [53, 54, 55]. This study examined the correlations between water intake and changes in ANG II, AVP and formaldehyde. However, we did not perform functional experiments, for example, we did not use AVP antagonists to block the action of AVP and subsequently examine changes in FA in the mice. Such functional experiments and the identification of the receptors used by formaldehyde to increase the levels of ANG II and AVP should be investigated in future work. Adipocytes, vascular endothelial cells, and smooth muscle cells are rich in SSAO, which generates formaldehyde. SSAO is expressed and activated in response to oxidative stress [56, 57, 58]. According to Van Kempen and colleagues [59], the administration of AVP to mice stimulates oxidative stress and causes hypertension, which increases the levels of SSAO. Endogenous formaldehyde levels are elevated through the deamination of methylamine, which is catalyzed by SSAO [60].

The responses of the endocrine and autonomic control systems are triggered by central and peripheral osmoreceptors to stimulate thirst [53]. As mentioned above, although the injection of formaldehyde did not significantly elevate the serum osmotic pressure compared with the controls (P=0.092), an effect of increased osmotic pressure on water intake could not be excluded. Furthermore, the administration of formaldehyde increased the serum ALD levels. Aldosterone plays a role in the regulation of blood pressure primarily via action on the distal tubules and collecting ducts of the nephron and increasing the reabsorption of ions and water in the kidney to conserve sodium, secrete potassium, increase water retention and increase the blood volume and pressure [61]. ALD also appears to affect the water intake behavior of mice, but the mechanism requires further clarification.

Formaldehyde is seriously toxic to human health and is particularly harmful to our central nervous system. To prevent the accumulation of endogenous formaldehyde in our bodies, we suggest that Chinese elderly people should establish regular water intake habits based on the instructions in the Chinese DRIs Handbook [41]. Specifically, elder people should drink a glass of water after they wake up in the morning. There are several reasons for our suggestion: 1) formaldehyde is continuously produced in our bodies [62, 63]; 2) endogenous formaldehyde gradually accumulates as we age [24, 45]; 3) As mentioned above, endogenous formaldehyde levels are greater in the morning than at noon and during the evening; 4) water deprivation led to the accumulation of endogenous formaldehyde in the participants’ bodies until they drank water; 5) formaldehyde is easily dissolved in water, and its removal from the blood depends upon renal function [64]; and 6) patients with Alzheimer’s disease exhibit high concentrations of endogenous formaldehyde.

Based on the instructions of the China Nutrition Association [41], in addition to water from food, adequate water intake for Chinese adults is 1.7 L/day for males and 1.5 L/day for females. It is suggested that healthy adults (400-500 ml for males and 350-400 ml for females) should drink warm boiled water, mineral water or green tea (according to one’s preference) after they wake up before breakfast because the concentration of endogenous formaldehyde peaks in the morning. Formaldehyde dissolves well in water. Drinking water aids our elimination of endogenous formaldehyde through the kidneys. The remaining water (1,000-1,200 ml) can be consumed during the day and evening according to one’s own habits. Regular water intake contributes to the elimination of endogenous formaldehyde and other harmful metabolic products.

In conclusion, chronic dehydration in elderly people and AD patients may result from their diminished perception of thirst and memory loss. Dehydration decreases the body’s ability to eliminate metabolic products and results in the accumulation of cytotoxic compounds, including endogenous formaldehyde, which not only affects water intake behavior but also cognitive function. The interaction between dehydration and cognitive impairment creates a vicious cycle. However, regular water intake can reduce the accumulation of formaldehyde, particularly when it is consumed in the morning.

## 

Supplementary Figure 1. **Changes in the body weights and serum osmolality of the water-deprived mice**. The brain formaldehyde concentrations (**A**) were significantly increased, and the average body weights (**B**) were significantly decreased following water deprivation for 3 days. The data were shown as the means ± SE; *, *P* < 0.05; **, *P* < 0.01

Supplementary Figure 2. **Changes in the body weights and serum osmolality of mice at different ages**. The animals’ body weights increased as they aged (**A**), but there were no significant changes in their serum osmolality levels (**B**) under the rearing conditions. The data are shown as the means ± SE; *, *P*<0.05; **, *P*<0.01.

Supplementary Figure 3. **Changes in the body weights, serum osmolality and ALD levels in mice injected with formaldehyde**. The conditions were the same as Figure 4, except that the body weights (**A**), serum osmolality (**B**) and ALD levels (**C**) were detected as described in the Materials and Methods. The data are shown as the means ± SE; *, *P* < 0.05.

Supplementary Figure 4. **Open field text after FA injected**. The conditions were the same as Figure 4. The number of crossing square (**A**), the vertical frequency (**B**), the number of grooming (**C**), and the time spending in central field (**D**) were detected as described in the Materials and Methods. The data are shown as the means ± SE.

Supplementary Figure 5. **Uric formaldehyde concentrations in the Alzheimer’s patients and normal elderly participants**. The conditions are described in Supplementary Tables 5 and 6, and the formaldehyde levels were determined as described in the Materials and Methods. The formaldehyde concentrations were compared between (**A**) the AD (n = 62) and normal (n = 69) participants; (**B**) the male AD (n = 22) and normal (n = 29) participants and (**C**) the female AD (n = 40) and control participants (n = 40). The data are shown as the means ± SD; *, *P*<0.05; **, *P*<0.01; ***, *P*<0.001.

Supplementary Figure 6. **A putative vicious cycle between dysmetabolism of formaldehyde and AVP in decreased water intake**. The cycle may occur when either formaldehyde or AVP abnormally increases.

**Supplementary Table 1:** Physical examination results for the participants.

Index	ALT(U/L)	GLU(mmol/L)	Urea(mmol/L)	CREA(μmol/L)	UA(μmol/L)
Normal value *	0-40	3.90-6.10	2.89-8.20	44-120	140-420
Results	12.48±7.96	4.68±0.28	4.25±0.95	81.07±9.00	329.53±83.89

**Index**	**TG(mmol/L)**	**TC(mmol/L)**	**HDL(mmol/L)**	**LDL(mmol/L)**	
Normal value *	0.4-1.86	0-6.19	1.2-1.68	2.07-3.10	
Results	1.31±0.41	4.56±0.78	1.46±0.41	2.29±0.53	

* Referred to Diagnostics published by People’s Medical Publishing House, 2013. [65] Examined results were shown in mean ± SD. ALT: alanine aminotransferase; GLU: blood glucose; CREA: creatinine; UA: uric acid; TG: triglyceride; TC: total cholesterol; HDL: high density lipoprotein; LDL: low density lipoprotein

**Supplementary Table 2:** Body weight during experiment.

Day	The first day	The second day	The third day
body weight (kg)	62.11±2.83	61.82±2.79	61.82±2.82

Body weights were shown in mean ± SE. No significant changes were shown during the trial.

**Supplementary Table 3:** The recipes in the experiment.

	Breakfast	Lunch	Supper
Time	8:00-8:30	12:30-13:00	18:00-18:30
Diet	Chinese buns Preserved vegetable, shepherd’s purse), millet congee	Rice, plain vegetables (oilseed rape, cabbage, Chinese cabbage), one egg	Rice, Plain vegetables (oilseed rape, cabbage, Chinese cabbage), one egg

Daily food consumption was based on the participant’s own habit and the instruction of the Chinese DRIs Handbook [41], to prevent someone from too hungry or over intake.

**Supplementary Table 4:** **A**. Ages, MMSE scores, and concentrations of endogenous formaldehyde of AD patients and normal participants. **B**. Ages, MMSE scores, and concentrations of endogenous formaldehyde of AD patients and age-matched normal participants.

	Male		Female		Total	
	AD	Norm	AD	Norm	AD	Norm
No	22	29	40	40	62	69
A	79.25 ± 7.53	69.39 ± 5.84	82.00 ± 5.46	66.00 ± 6.19	81.05 ± 6.36	67.33 ± 6.19
S	6.60 ± 5.81	28.72 ± 1.31	5.10 ± 5.26	28.85 ± 1.21	5.55 ± 5.41	28.80 ± 1.17
FA	1.59 ± 0.87	1.21 ± 0.37	1.83 ± 0.83	1.04 ± 0.36	1.71 ± 0.86	1.11 ± 0.37
P	0.039		0.001		0.001	

	Male		Female		Total	
	AD	Norm	AD	Norm	AD	Norm
No	11	12	10	12	21	24
A	74.09 ± 3.21	75.17 ± 2.95	73.70 ± 1.88	73.83 ± 2.55	73.90 ± 2.61	74.5 ± 2.78
S	4.90 ± 5.43	28.00 ± 0.95	4.80 ± 4.04	27.92 ± 1.44	4.85 ± 4.70	27.96 ± 1.19
FA	1.52 ± 0.59	1.09 ± 0.26	2.29 ± 1.01	1.06 ± 0.26	1.84 ± 0.92	1.11 ± 0.32
P	0.043		0.01		0.01	

Data show in mean ± SD, where A, S, FA, P and Norm represent age, scores of MMSE, formaldehyde concentration (FA/Urc ratio), P values and normal participants

**Supplementary Table 5:** Approximate water intake (average volume) of the participants based on their own habits on trial day.

Number	Gender	Between breakfast and lunch (ml)	Between lunch and supper (ml)	Between supper and sleep (ml)	In total (ml)
1	F	400	400	250	1050
2	M	500	500	500	1500
3	F	400	400	200	1000
4	F	250	250	100	600
5	F	300	250	250	800
6	M	400	400	400	1200
7	M	600	600	600	1800
8	M	600	400	600	1600
9	F	300	200	400	900
10	M	200	400	400	1000
11	F	400	400	400	1200
12	M	200	150	150	500
13	F	250	400	200	850
14	F	400	400	400	1200
15	F	400	400	200	1000
16	M	400	400	400	1200
17	F	400	200	400	1000
18	M	300	400	200	900
19	M	400	400	400	1200
20	F	400	200	200	800
Average		375	358	333	1066

**Supplementary Table 6:** Concentrations of endogenous formaldehyde of AD patients and normal participants in female.

AD patients					Normal participants				
No	FA[μM]	G	A	S	No	FA[μM]	G	A	S
1	11.051323	F	83	16	1	7.809922	F	61	29
2	17.756591	F	74	9	2	9.495039	F	75	28
3	11.86835	F	86	3	3	9.421235	F	69	29
4	14.645642	F	85	8	4	8.409743	F	67	26
5	13.578474	F	76	6	5	11.2525	F	60	29
6	9.441871	F	84	0	6	5.158226	F	74	29
7	9.9753338	F	82	0	7	9.905576	F	61	29
8	15.142931	F	86	0	8	7.901044	F	65	29
9	8.2721696	F	82	12	9	5.488889	F	74	26
10	11.785239	F	84	0	10	7.090734	F	58	29
11	9.1878449	F	74	0	11	10.97525	F	65	28
12	11.026722	F	85	5	12	11.48897	F	74	30
13	8.1637291	F	74	9	13	11.20549	F	71	29
14	15.828454	F	88	11	14	18.29936	F	56	30
15	16.644024	F	86	3	15	8.877414	F	60	30
16	14.724626	F	89	4	16	6.235591	F	61	30
17	15.258979	F	92	0	17	6.364749	F	57	30
18	29.788072	F	70	0	18	5.803205	F	58	30
19	11.249349	F	84	0	19	19.52005	F	75	29
20	7.7219552	F	85	3	20	8.528704	F	60	30
21	8.2186777	F	82	9	21	9.271846	F	80	25
22	26.815748	F	86	3	22	15.00439	F	63	29
23	10.943206	F	85	7	23	9.079647	F	65	29
24	17.630347	F	74	9	24	5.598543	F	63	30
25	17.283337	F	74	3	25	4.720717	F	69	30
26	11.672024	F	85	5	26	7.215469	F	61	30
27	9.029709	F	86	0	27	6.031566	F	75	28
28	19.56695	F	74	3	28	12.57158	F	65	30
29	8.869459	F	71	9	29	12.71254	F	69	29
30	10.84791	F	82	12	30	7.536957	F	59	30
31	10.49515	F	85	10	31	7.406109	F	67	29
32	9.751272	F	83	16	32	9.566842	F	70	27
33	18.87941	F	83	16	33	9.449732	F	60	29
34	15.39848	F	84	0	34	11.67828	F	72	28
35	12.35077	F	82	0	35	5.499434	F	74	29
36	11.23963	F	82	13	36	10.85604	F	61	29
37	13.11016	F	84	0	37	7.142598	F	66	29
38	11.30942	F	81	0	38	11.00442	F	63	29
39	18.99518	F	76	0	39	9.29877	F	72	27
40	24.89504	F	92	0	40	6.774013	F	65	29

Raw data in anonymity, where A, S, FA represent age, scores of MMSE, and formaldehyde.

**Supplementary Table 7:** Concentrations of endogenous formaldehyde of AD patients and normal participants in male.

AD patients					Normal participants				
No	FA[μM]	G	A	S	No	FA[μM]	G	A	S
1	16.82044	M	88	4	1	13.82214	M	64	30
2	10.90323	M	78	12	2	13.59417	M	74	30
3	8.948305	M	70	15	3	12.72268	M	64	28
4	16.65819	M	77	0	4	12.61392	M	69	28
5	8.666764	M	72	0	5	9.984478	M	73	28
6	11.03918	M	78	0	6	7.911322	M	79	29
7	9.747851	M	83	5	7	11.32809	M	76	27
8	16.13346	M	76	6	8	9.924351	M	59	27
9	19.31118	M	72	7	9	11.88364	M	66	29
10	13.70019	M	70	0	10	8.464368	M	67	30
11	17.47359	M	71	5	11	9.251291	M	65	30
12	11.66296	M	86	16	12	10.13201	M	78	28
13	5.222967	M	88	8	13	14.4900146	M	64	29
14	28.72713	M	88	4	14	10.140191	M	74	27
15	17.63035	M	74	9	15	11.8311113	M	67	28
16	11.06712	M	83	3	16	6.2413308	M	65	30
17	15.47421	M	64	0	17	9.95427717	M	73	27
18	21.4961	M	88	4	18	9.32465406	M	72	28
19	7.903619	M	88	15	19	8.80549114	M	70	28
20	9.575305	M	87	10	20	7.90389413	M	65	30
21	11.92712	M	77	0	21	8.85725905	M	76	29
22	9.351719	M	87	17	22	9.67120231	M	65	30
23					23	8.70657156	M	79	28
24					24	9.87167933	M	59	30
25					25	9.00041766	M	66	30
26					26	14.4446765	M	67	30
27					27	8.200488	M	65	29
28					28	7.17480189	M	67	29
29					29	9.25722388	M	78	27

**Supplementary Table 8:** Concentrations of endogenous formaldehyde of AD patients and normal participants in male and female groups.

Female group			Male group		
No	Normal	AD patients	No	Normal	AD patients
1	0.971866849	1.431982266	1	1.393009826	1.707832267
2	1.088630933	3.058054148	2	2.003119428	0.910081382
3	1.805353071	1.245301917	3	2.200394327	0.931726885
4	1.107273601	2.16587432	4	1.30673573	1.888683673
5	1.801120448	1.944643572	5	1.047634227	1.059118172
6	0.64582772	1.338133646	6	0.700457922	1.482167025
7	1.573187644	1.62862593	7	1.242932851	1.279329484
8	0.743068184	1.884386651	8	1.431362371	0.658508571
9	0.788860161	0.87926973	9	1.430815725	2.057993286
10	0.826907755	1.636157037	10	0.888134725	1.100770529
11	1.157543638	0.776426665	11	0.854307046	2.06129409
12	1.026121556	1.875292806	12	1.168224374	1.181238669
13	1.407282889	1.405963844	13	1.754982695	0.462434548
14	1.390898795	1.774888261	14	1.352566496	4.727967413
15	0.726139135	2.272066566	15	1.267460636	2.60726856
16	0.859844319	1.970508628	16	1.027210467	1.220862659
17	1.082440306	2.281375304	17	1.692904281	1.720982039
18	0.839948618	4.121490355	18	1.014928333	1.578042872
19	1.541076856	1.905216115	19	0.95081429	0.935062881
20	0.784032359	0.882862307	20	0.888727063	1.206261653
21	0.982968036	1.490916585	21	0.910631681	1.12429844
22	1.650738765	4.73818326	22	1.52410406	1.066208984
23	1.147361724	1.436210549	23	0.935184915	
24	0.367974169	2.200629991	24	0.77336984	
25	0.386136927	2.734272644	25	1.043647688	
26	0.621326875	1.212237	26	1.448596147	
27	0.882388413	1.275292564	27	0.937270727	
28	1.062372079	3.27315992	28	0.818219356	
29	1.35832247	1.311662082	29	1.021152116	
30	0.71210856	1.537402211	30		
31	0.74162306	1.227431144	31		
32	1.067458847	1.153655368	32		
33	1.72483998	1.735558926	33		
34	1.380901029	1.636743197	34		
35	0.878784596	1.344301497	35		
36	1.097911589	1.079436255	36		
37	0.816997198	1.597339019	37		
38	0.904992969	1.162743022	38		
39	1.061636735	2.062003908	39		
40	0.660441171	2.618876499	40		
