# Supplementary figures and images for: Brain Formaldehyde is Related to Water Intake behavior
Source: Aging Dis. 2016 Oct 1;7(5):561–84. doi: 10.14336/AD.2016.0323 (PMC5036952; doi:10.14336/AD.2016.0323)

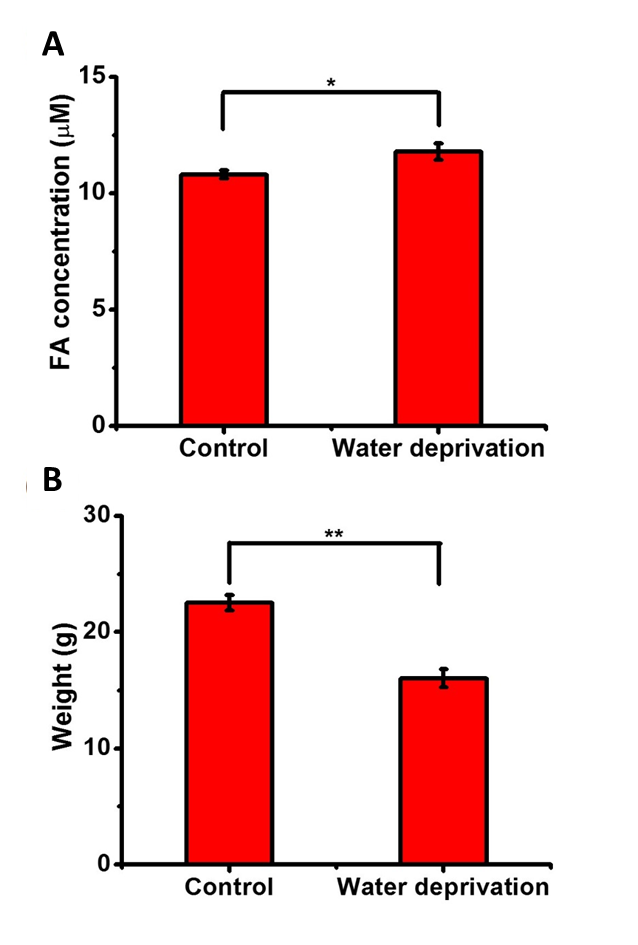

Supplement: Supplementary Figure 1. — Changes in the body weights and serum osmolality of the water-deprived mice. The brain formaldehyde concentrations (A) were significantly increased, and the average body weights (B) were significantly decreased following water deprivation for 3 days. The data were shown as the means ± SE; *, P < 0.05; **, P < 0.01 [file ad-7-5-561-g6.tif]

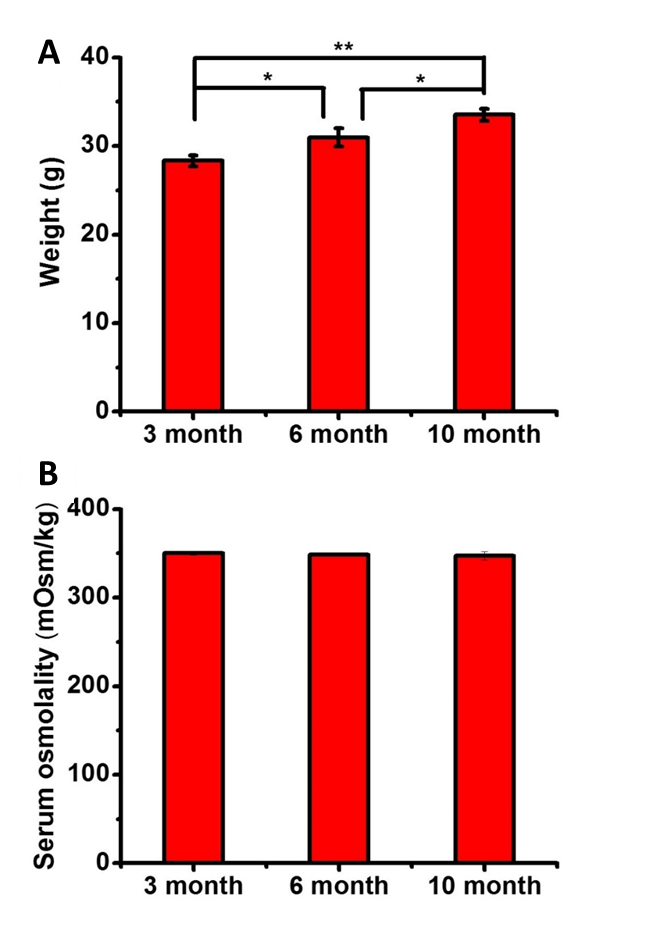

Supplement: Supplementary Figure 2. — Changes in the body weights and serum osmolality of mice at different ages. The animals’ body weights increased as they aged (A), but there were no significant changes in their serum osmolality levels (B) under the rearing conditions. The data are shown as the means ± SE; *, P<0.05; **, P<0.01. [file ad-7-5-561-g7.tif]

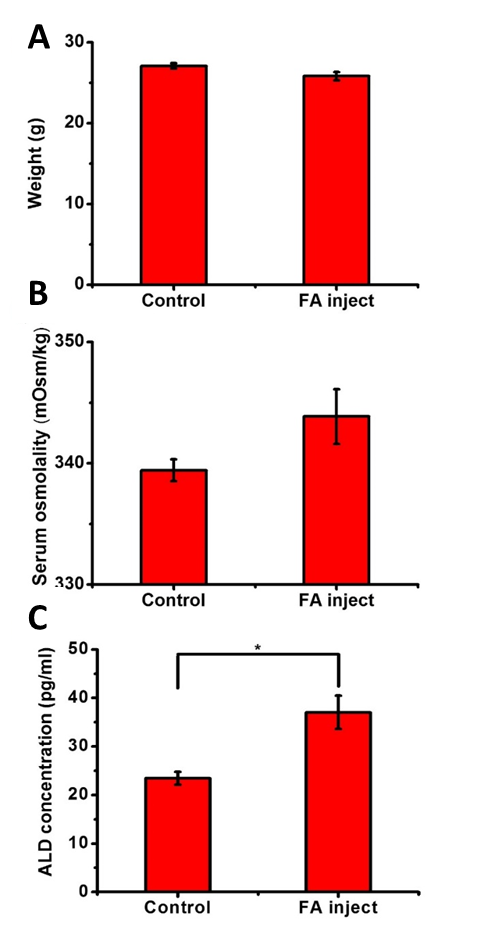

Supplement: Supplementary Figure 3. — Changes in the body weights, serum osmolality and ALD levels in mice injected with formaldehyde. The conditions were the same as Figure 4, except that the body weights (A), serum osmolality (B) and ALD levels (C) were detected as described in the Materials and Methods. The data are shown as the means ± SE; *, P < 0.05. [file ad-7-5-561-g8.tif]

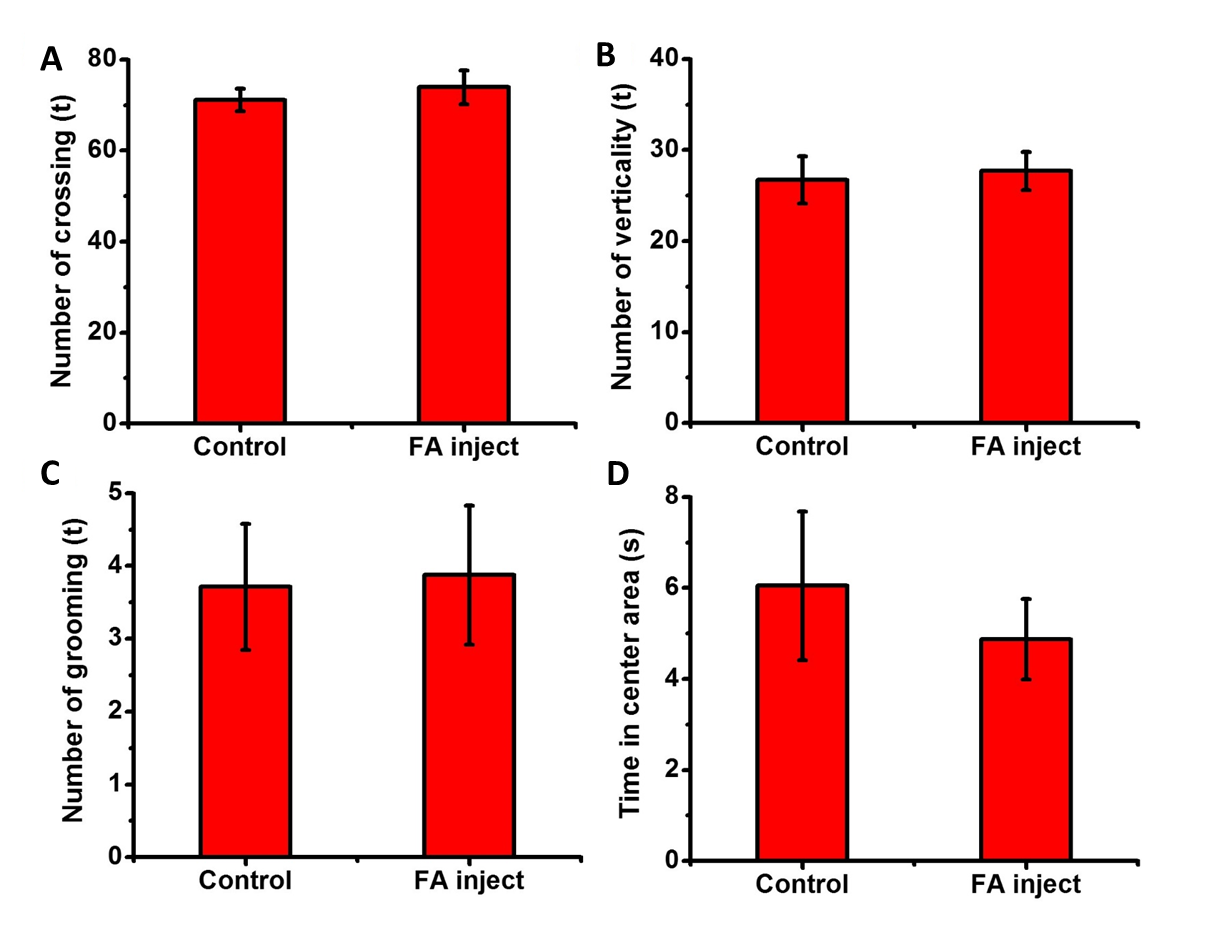

Supplement: Supplementary Figure 4. — Open field text after FA injected. The conditions were the same as Figure 4. The number of crossing square (A), the vertical frequency (B), the number of grooming (C), and the time spending in central field (D) were detected as described in the Materials and Methods. The data are shown as the means ± SE. [file ad-7-5-561-g9.tif]

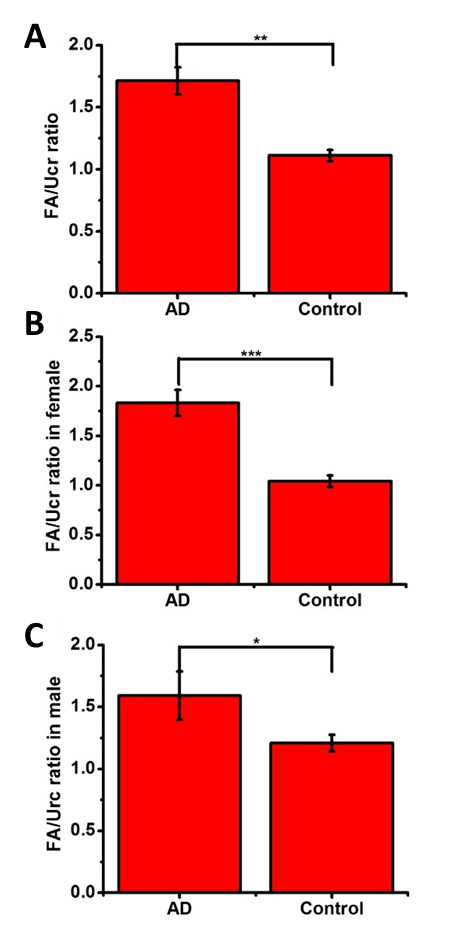

Supplement: Supplementary Figure 5. — Uric formaldehyde concentrations in the Alzheimer’s patients and normal elderly participants. The conditions are described in Supplementary Tables 5 and 6, and the formaldehyde levels were determined as described in the Materials and Methods. The formaldehyde concentrations were compared between (A) the AD (n = 62) and normal (n = 69) participants; (B) the male AD (n = 22) and normal (n = 29) participants and (C) the female AD (n = 40) and control participants (n = 40). The data are shown as the means ± SD; *, P<0.05; **, P<0.01; ***, P<0.001. [file ad-7-5-561-g10.tif]

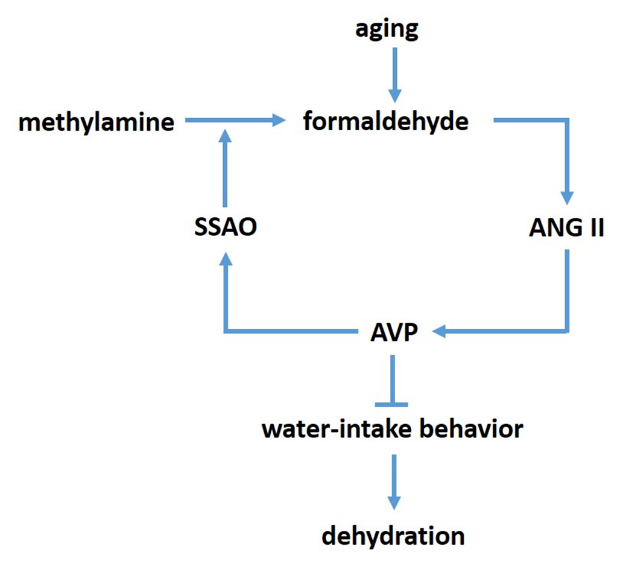

Supplement: Supplementary Figure 6. — A putative vicious cycle between dysmetabolism of formaldehyde and AVP in decreased water intake. The cycle may occur when either formaldehyde or AVP abnormally increases. [file ad-7-5-561-g11.tif]
